# Response to novel feed in dairy calves is affected by prior hay provision and presentation method

**DOI:** 10.1371/journal.pone.0284889

**Published:** 2023-05-03

**Authors:** Chelsea R. Morrow, Blair C. Downey, Cassandra B. Tucker

**Affiliations:** 1 Center for Animal Welfare, Department of Animal Science, University of California, Davis, Davis, CA, United States of America; 2 School of Veterinary Medicine, University of California, Davis, Davis, CA, United States of America; 3 Animal Behavior Graduate Group, University of California, Davis, Davis, CA, United States of America; Tokat Gaziosmanpasa Universitesi, TURKEY

## Abstract

Animals raised in environments that prevent natural foraging opportunities may have difficulty adapting to novelty, such as feeding and management changes. Our objective was to evaluate how early provision and presentation of forage in dairy calves affected response to novel TMR (total mixed ration; grain and alfalfa) at weaning. Holstein heifer calves were housed individually in a covered outdoor hutch with an attached uncovered wire-fenced pen on sand bedding. Calves were fed a diet of starter grain and milk replacer (5.7–8.4L/d step-up) via a bottle (Control, n = 9) or given additional access to mountaingrass hay presented either in a bucket (Bucket, n = 9), or PVC pipe feeder (Pipe, n = 9). Treatments were applied from birth through 50 d of age, when step-down weaning began. All calves had 3 buckets and a pipe feeder provided in their uncovered pen area. On d 50, each calf was briefly blocked inside their hutch. TMR was put in the 3^rd^ bucket that previously contained hay (Bucket) or was empty (Control, Pipe). The calf was released from the hutch and video-recorded for 30 min. Neophobia towards TMR was affected by prior experience with presentation: Bucket calves began eating TMR faster than Pipe and Control (P≤0.012) and showed the fewest number of startle responses (P = 0.004). Intake was similar across groups (P = 0.978), suggesting this apparent neophobia was transient, but Control calves took longer to eat than Bucket (P<0.001) and Pipe (P = 0.070) calves and were less likely to give up on eating to lie down instead. These results suggest that previous experience with hay improves processing ability when presented with novel TMR. Overall, response to a novel feed is affected by both early life experience, such as opportunities to process forage, and the presentation of the feed itself. Calves also appear motivated to access forage, evidenced by transient neophobia, high intake, and persistence in feeding by naïve calves.

## Introduction

Complexity in early life can improve behavioral flexibility and adaptability to new situations. For example, finches reared in conditions where food delivery was unpredictable were better able to solve novel challenges as adults [1], while mink raised with environmental enrichment solve cognitive tasks better in later life [2]. Dairy calves have been shown to consume more novel feed and have better learning ability when raised in complex social housing as opposed to individual housing [3, 4]. A common new situation, especially experienced by young developing animals, is the introduction and adaptation to novel feed. This can elicit neophobia, or a heightened reaction to novel stimuli, in inexperienced animals. Food neophobia may stem from an evolutionary drive to avoid potentially harmful foods [as reviewed by 5] but can be problematic if it impedes acquisition of nutrients or transition to new, necessary diets [6]. Food neophobia can include both object neophobia, or fear of appearance of the food or how it is presented, and dietary wariness, or hesitation to consume the feed [7]. This fear can be expressed via startle responses [8, 9], long latencies to approach or eat novel feed [10–12], and low intake or slow consumption rates [6, 13, 14]. Early exposure to social companions and diverse feed can improve adaptation to novel foods (e.g. lambs, [14]), as can additional exposure to the same object or diet (e.g. cattle, [13], ravens [15]). Thus, the skills and familiarity gained from early life complexity and prior experience with similar feed may reduce the intensity of food neophobia.

Dairy cattle in the United States are often raised in intensive environments lacking complexity, which influences adaptability to changes, especially in young animals. Calves in these settings are typically reared individually and fed grain and limited milk [16], restricting opportunities to perform natural behaviors including sucking, chewing, and ruminating. This barren environment is known to cause welfare problems, including hunger [17, 18], abnormal repetitive behaviors [19–21], and impaired performance on cognitive tasks [4, 22, 23], and may influence their behavioral flexibility and adaptation to new scenarios, including feed provision. Dairy calves are often not fed forage, like a total mixed ration (TMR, grain and forage mix often provided to adult cattle), until 1–2 months of age, which is around the time weaning off of milk occurs [16]. The transition from liquid- to solid-based diet is known to be stressful, indicated by increased vocalization and locomotion [24], as calves struggle to adapt quickly to the new feed. They often experience a growth check, or decreased weight gain, during this period as a result [3, 6, 24]. These responses may be exacerbated by neophobia, especially since forage requires more oral processing (chewing and ruminating) and motor skills to break down than grain [25–27], and can be presented in several distinct ways, including buckets, hay racks, or pasture [16, 19, 28, 29]. These multiple facets of novelty, particularly after experiencing unstimulating environments, could lead to heightened neophobia that calves must quickly overcome in order to avoid hunger.

Difficulty adapting to a diet change may be improved by early life forage provision. Calves typically consume forage like hay within 1 d of birth when given the chance [19, 30] and seem motivated to access it. They will selectively consume 15–20% of their diet in the form of hay when given the opportunity [19, 26, 31] and perform fewer abnormal behaviors when it is provided [19, 32, 33]. Access to hay seems to improve processing skills broadly, leading to increased grain intake, overall dry matter intake, and average daily gain as a result [26, 27, 34], and can improve TMR intake after weaning [19]. This may be because forage provides more practice chewing and ruminating on larger, longer, rigid, and more fibrous particles compared to grain. Early experience processing hay may thus improve behavioral response to novel forage introduction at weaning.

Our objective was to evaluate how early provision and presentation of forage in dairy calves from birth affected response to novel forage provided in a bucket at weaning. We expected that neophobia, including latency to begin consuming novel TMR and startle reactions, would be reduced by prior experience with hay and familiarity with method of forage presentation. Calves inexperienced with hay would be most neophobic, followed by those who were previously fed hay in a pipe feeder (designed to mimic more naturalistic feeding behavior; [34]). Those with experience with hay in a bucket would be least neophobic. As forage seems important for young calves, we expected that those inexperienced with hay would stay engaged with TMR for longer, while those familiar with consuming hay would be more likely to cease eating sooner and turn to other behaviors, like rest. Lying down after a meal has been used as a proxy for satiety [35], and switching to this behavior could indicate that calves are no longer motivated to continue feeding. We also expected that inexperienced calves would be less efficient at processing more rigid and fibrous forage, evidenced by them taking longer to consume the same amount of TMR as experienced calves.

## Materials and methods

All data are publicly available in the Dryad repository (https://doi.org/doi:10.25338/B8GK93, [36]) along with RMarkdown files for all analyses and figures, and a supplemental table providing test statistics, CI, and df.

### Experimental setup

This experiment was run at the UC Davis Dairy Facility. All procedures were approved by the Animal Care and Use Committee (protocol #20466). We enrolled all healthy female Holstein calves born between June-September 2019 (n = 27 calves). On the day of birth, each calf was placed in an individual outdoor plastic hutch (2 x 1.5 m, length x depth) with an attached wire-fenced pen (2 x 1.5 x 0.9 m, length x depth x height). The enclosures were bedded with sand ~12–17 cm deep that was spot-cleaned daily and topped-up as needed. The bedding was covered with perforated rubber mats from d 0–5 ± 1 to limit unintentional inhalation of sand particulate (see [34] for more details). All calves received colostrum 2x/d for 5 d per farm protocol via a bottle and rubber teat (Connewango). From 5–9 d of age, calves were fed 1.9 L of milk replacer (26% CP, 16% fat, 15% total solids, mixed as indicated at a rate of 142 g/L of hot water; Calva Products Inc.,) at each of 2 daily meals at approximately 09:15 h and 16:15 h. From 10–23 d of age, calves received 2.4 L of milk replacer at each of the 2 meals, and from 24–49 d of age, 2.8 L of milk replacer per meal. Weaning began at 50 d when the 09:15 h meal was removed and calves received TMR (89.7% DM; alfalfa, almond hulls, cottonseed, corn, barley, beet pulp; S1 Fig, [36]; chemical composition and particle size reported in [34]) for the first time, as per farm protocol. Calves were fully weaned at 60 d when the 16:15 h meal was removed.

### Feeding treatments

Calves were assigned to 1 of 3 treatments (n = 9/treatment) at birth as part of a separate experiment [34]: Control, access to hay in a bucket (Bucket), or access to hay in a pipe feeder (Pipe). Calves were allocated to 1 of 3 treatments based on birth order, with each treatment represented in a given similarly aged cohort of 3 calves using a random number generator. Adjustments were made to balance birth weight across treatments (Control: 38.6; Bucket: 38.6, Pipe: 38.8 kg). Calves in the Control treatment had access to ad libitum water and grain (Starter Calf Feed 901033, Associated Feed & Supply Co; S1 Fig, [36]; chemical composition reported in [34]) by bucket. Calves in the Bucket treatment received ad libitum water, grain, and chopped (19 ± 4 cm, mean ± SD) mountaingrass hay (mix of orchard, *Dactylis glomerata*, and fescue, *Festuca arundinacea;* Higby’s Country Feed; S1 Fig, [36]; chemical composition and particle size reported in [34]) by bucket from birth. Calves in the Pipe treatment received ad libitum water, grain, and chopped mountaingrass hay in a 56x10.2-cm (length x diameter) polyvinyl chloride (PVC) pipe feeder. The pipe feeder had 4 equally spaced 6.35-cm holes cut into it that were sanded down until smooth and was fitted with 2 removable knockout end caps. Pipes were mounted on the wire-fenced pen 0.8 m from the ground using a 10.2-cm vent pipe hanger in all pens (see [34, 37] for more details, including photos and videos). All calves had 3 buckets available at all times in the attached wire-fenced pen (left to right): grain, water, hay (Bucket) or empty bucket (Control and Pipe). All pens contained a pipe feeder filled with hay (Pipe) or kept empty (Control and Bucket).

### Data collection

On d 50, before the morning milk feeding was withheld to start step-down weaning, the calf was briefly confined inside her hutch with an opaque barrier while the novel test was set up. A video camera (Panasonic HC-V550) was positioned directly in front of the hutch, so that the inside of the hutch, the outside yard and all 3 feed buckets were visible. The 3rd bucket, which either previously held hay (Bucket calves) or was empty (Control and Pipe calves) during the preweaning period, was filled with TMR and replaced in the pen. Buckets were filled with pre-weighed bags of TMR (minimum 645 g) so that the feed would be visible over the rim of the bucket. The test began when the opaque barrier was removed (between 08:15–08:40) and the calf was released from the hutch. Novel feed tests were conducted for 30 min, similar to other tests for novelty in calves (e.g. novel feed, [3]; novel environment, [38]). At the end of the 30-min period, the TMR bucket was removed. Intake during the test period was calculated by subtracting refusals from the initial provision (GBK16a Bench Check Weighing Scale 8000 g limit / 0.1 g readability, Adam Equipment Inc.). All calculations were done on a dry matter basis as described in [34].

Videos were scored by 1 observer, blind to treatment, using Behavioral Observation Research Interactive Software (BORIS, http://www.boris.unito.it/; [39]) for continuous and point behavioral responses to TMR provision: retreat, startles, flinches, latency to begin eating TMR, eating TMR, and lying down (Table 1). Latency to eat TMR was calculated as the time from first orientation or proximity to the TMR bucket to the start of eating TMR. This starting point was used instead of the time of release from the hutch in order to minimize the effects of manual removal of the barrier which interfered with the environment and may have created a disturbance. The observer was trained before the start of video analysis by watching 13 video clips (5 min each, 13 unique calves) on 4 separate occasions; intra-rater reliability was calculated using the 3^rd^ and 4^th^ viewings (intraclass correlation coefficient = 1 for all behaviors; *irr* package version 0.84.1, [40]).

**Table 1:** Behaviors recorded from video using behavior sampling and continuous recording during the 30-min novel TMR test.

Behavior	Scoring Type	Scoring Definition
Retreat	Point	Calf takes at least 1 step away from bucket while oriented to the bucket
Startle	Point	Rapid retraction of the head towards the body and contraction of the ribcage (both elements required)
Flinch	Point	Rapid retraction of the head towards the body or contraction of the ribcage (only 1 of the elements required)
Latency to eat TMR	State	Time from first orientation (head facing TMR but not within 1 head length of the bucket) or first proximity to bucket (muzzle^1^ within 1 head length of bucket), whichever occurred first, to start of eating TMR
Eating TMR	State	Any part of the muzzle^1^ enters the TMR bucket for at least 1 s, mouth closes around TMR, or there are jaw movements within a head-length of the TMR bucket for at least 2 s
Latency to lie down	State	Time from removal of opaque barrier to start of lying down
Lying down	State	Weight of the calf is supported by the body, i.e. calf is in recumbent position

^1^Muzzle defined as from the bottom of the eyes to the end of the mouth.

### Statistical analysis

Some data were excluded. Video recordings for 1 Pipe and 1 Bucket calf were corrupted, and thus were not scored, leaving 25 calves for behavioral measures. One Control calf was only scored for 20 min due to a camera battery failure. This calf was excluded from lying down, leaving 24 calves for this analysis. Continuous behaviors are expressed as a proportion of the video duration. One Bucket calf was excluded from the TMR consumption analysis due to DM conversion producing a negative value, leaving 26 calves for this measure.

Statistical analyses were performed using R version 4.2.2 [41] on macOS Ventura 13.0 via RStudio version 2022.12.0+353 [42] with calf as the experimental unit. Model fit was checked for normality and homogeneity of variance using QQ plots and plots of residual vs. fitted values (*plot*, *boxplot*, *resid* functions in base R, *ggpubr* package version 0.6.0 [43]); unless stated otherwise, all model assumptions were met. All model fits were assessed with an ANOVA (type II SS; *car* package version 3.0.1–1 [44]) to obtain p-values. If significant (P < 0.05) treatment effects were detected, p values for individual pairwise comparisons were obtained using estimated marginal mean contrasts (*emmeans* package version 1.8.4–1 [45]). Results are reported as means and standard errors.

A beta regression model (*betareg* package v. 3.1–4 [46]) was used to test the effect of treatment on proportion of time spent eating TMR. Latency to begin eating TMR and amount of TMR consumed were both assessed with generalized linear models (*glm* function in base R) using a Gamma distribution. Due to rarity, counts of retreats, flinches, and startles were combined. The number of animals per treatment that did any of these 3 behaviors was analyzed with a chi-square test (*chisq*.*test* function in base R). The number of calves that laid down during the test period per treatment was also analyzed with a chi-square test.

## Results

**Fig 1:**
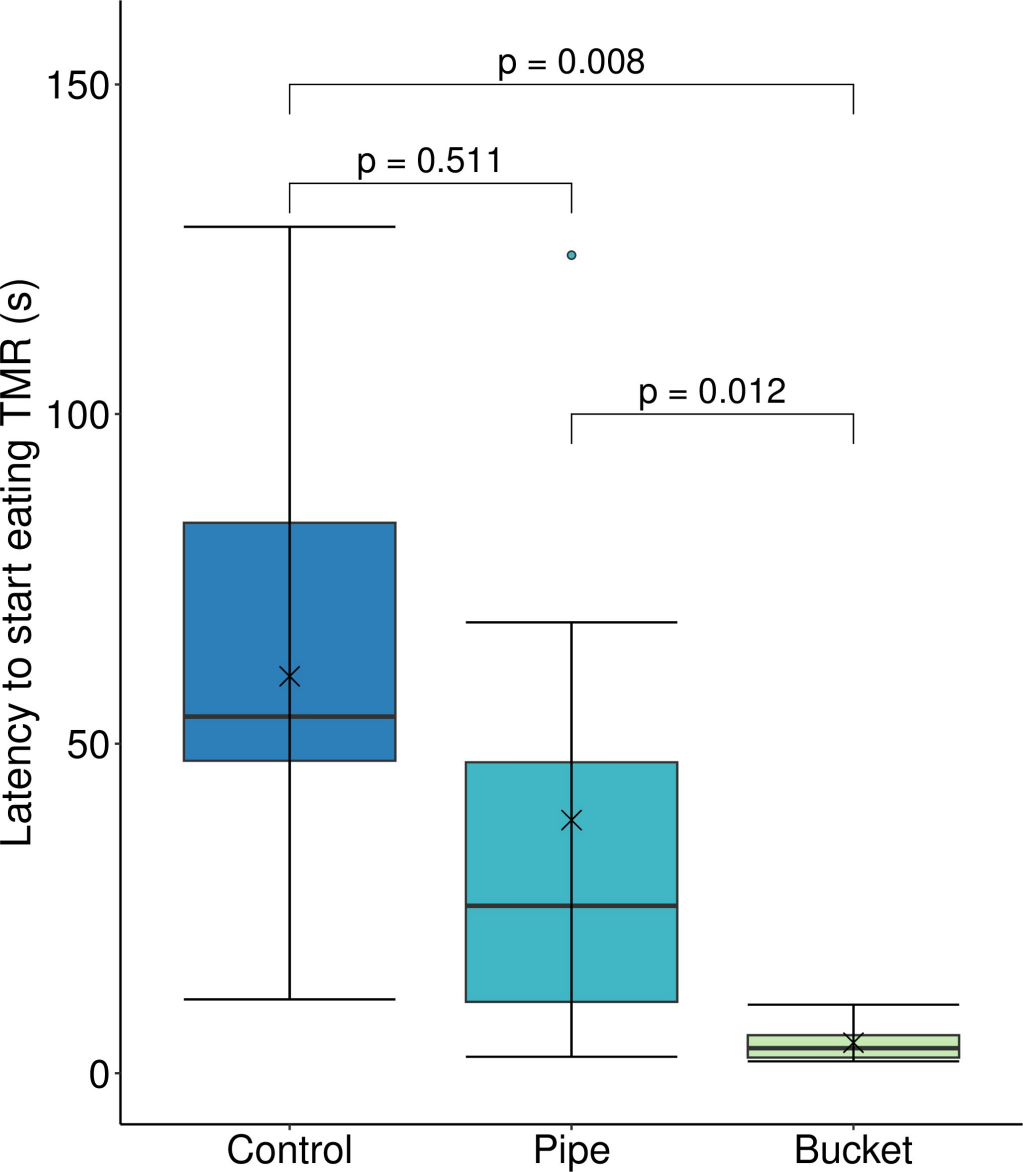
Latency to start eating novel TMR once oriented to, or in proximity of, the TMR bucket. Dairy calves with no prior hay experience (Control, n = 9), prior experience consuming hay from a pipe feeder (Pipe, n = 8), and prior experience consuming hay from a bucket (Bucket, n = 8) were evaluated for their response to the introduction of novel TMR at 50 d of age. Boxplots represent the median (black line within box) and first and third quartiles (25 and 75% of data). Whiskers extend to the lowest and highest values that are not outliers (values that are 1.5x the interquartile range); outliers (o) and means (x) are also presented.

Bucket calves began eating TMR within 5 ± 1 s (mean ± SE) of first orienting to or being in proximity of the bucket, which was significantly faster than both Control (60 ± 12 s, P = 0.008) and Pipe (38 ± 14 s, P = 0.012) calves (Fig 1; test statistics, df, and P-values reported in S1 Table, [36]). Control and Pipe calves had similar latencies to start eating TMR (P = 0.511). The total number of calves that performed any retreats, startles, or flinches differed by treatment (Control: 9, Pipe: 6, Bucket: 2; P = 0.004; test statistics, df, and P-values reported in S1 Table, [36]). A majority (86%) of startles, flinches, and retreats occurred within the first 200 s after the barrier was removed. For examples of how each treatment responded to novel TMR, see S1–S3 Videos, also available on Dryad [36].

**Fig 2:**
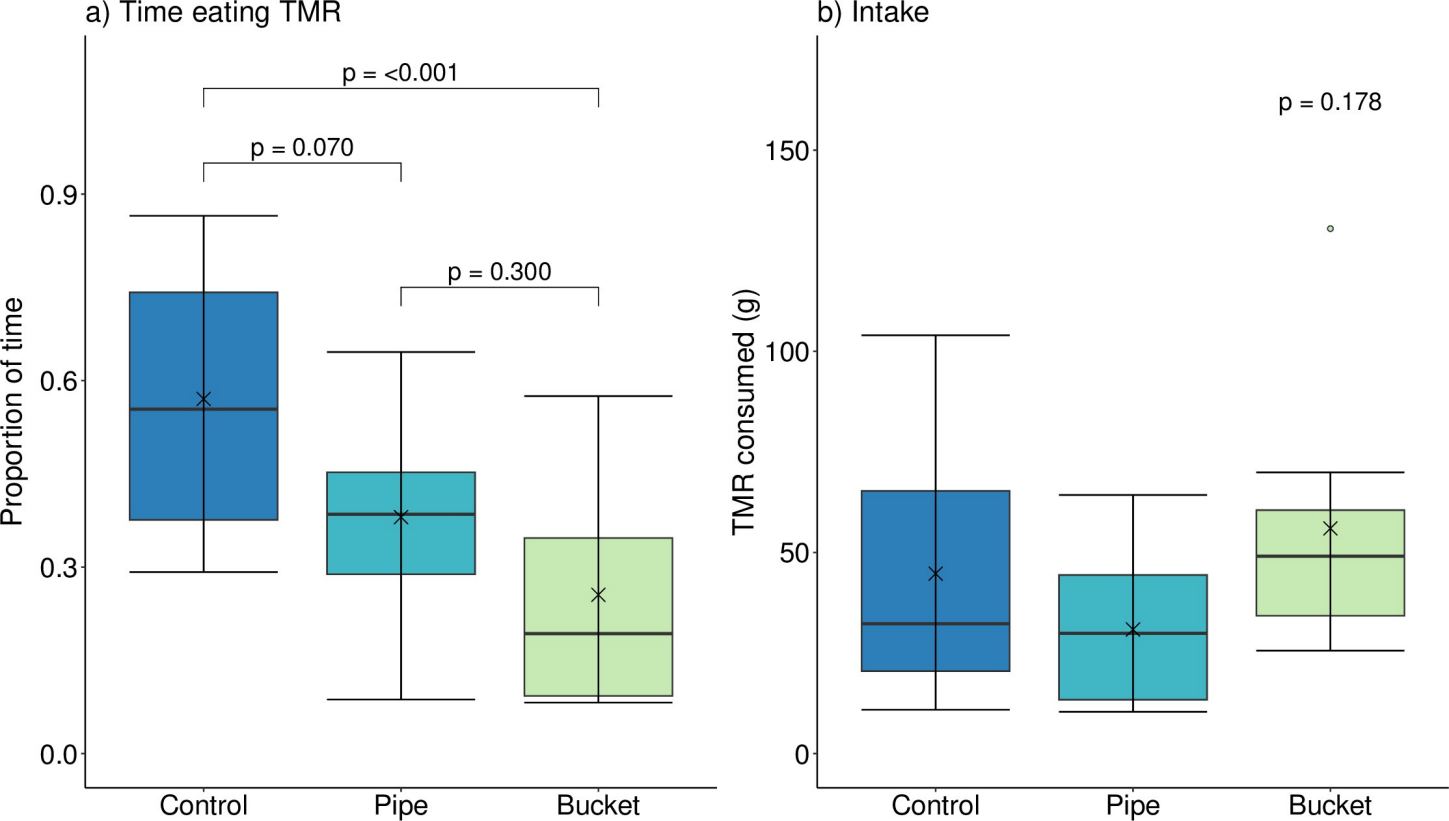
Proportion of time spent eating TMR (a) and total TMR intake (b) during a 30-min novel feed test. Dairy calves with no prior hay experience (Control), prior experience consuming hay from a pipe feeder (Pipe), or prior experience consuming hay from a bucket (Bucket) were evaluated for their response to the introduction of novel TMR at 50 d of age. Boxplots represent the median (black line within box) and first and third quartiles (25 and 75% of data). Whiskers extend to the lowest and highest values that are not outliers (values that are 1.5x the interquartile range); outliers (o) and means (x) are also presented.

**Fig 3:**
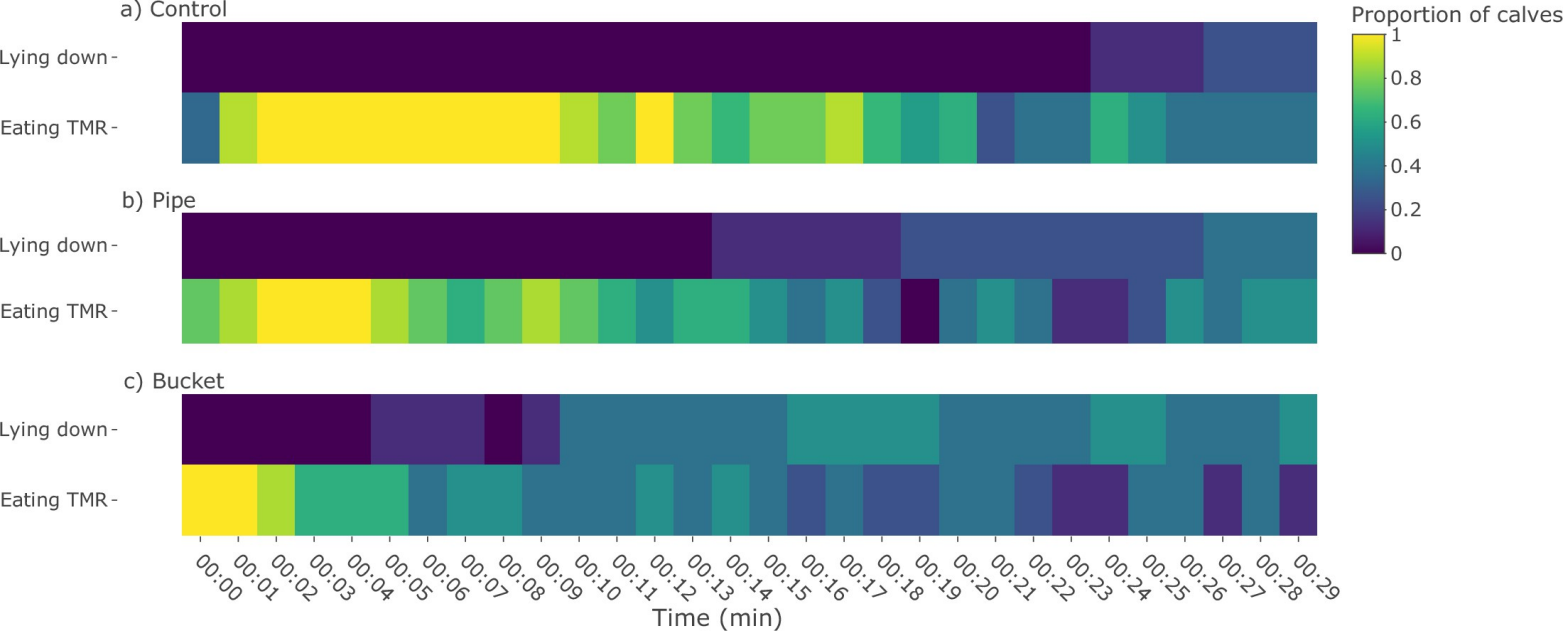
Proportion of calves eating TMR or lying down over the first 30 min following introduction of TMR. Dairy calves with no prior hay experience (Control), prior experience consuming hay from a pipe feeder (Pipe), or prior experience consuming hay from a bucket (Bucket) were evaluated for their response to the introduction of novel TMR at 50 d of age. Behavior was scored continuously and binned by minute. Bright yellow indicates that all calves in that treatment performed the behavior at that minute, while dark blue indicates no individuals performed the behavior.

Bucket calves spent significantly less time eating TMR than Control calves (P < 0.001, Fig 2; test statistics, df, and P-values reported in S1 Table, [36]). Pipe calves spent a similar amount of time eating as Bucket calves (P = 0.300) and tended to spend less time eating than Control calves (P = 0.070). All calves consumed 43.8 ± 30.1 g of TMR during the test (P = 0.178; Fig 2; test statistics, df, and P-values reported in S1 Table, [36]). Some calves laid down after eating TMR: 2 Control (after 1491–1643 s; Fig 3), 6 Pipe (after 891–1816 s), and 4 Bucket calves (after 320–655 s). There was no difference in the number of calves that laid down by treatment (P = 0.135). Not all calves laid down, indicating our test was likely too short to evaluate meaningful differences in this outcome.

## Discussion

Response to novel TMR was affected by familiarity with forage and method of feed presentation. Control calves took significantly longer to start eating TMR than Bucket calves and performed more startles, flinches, and retreats, while Pipe calves responded intermediately in both measures. These responses are commonly used as metrics of neophobia (e.g. increased latency to approach and touch an item: [11, 15, 47]; startles or retreats: [4, 15, 48]) and suggest that calves were responding to 2 types of novelty: new feed (experienced only by Control) and its presentation (both Control and Pipe), with Control calves facing compounded novelty compared to Pipe. This is consistent with evidence across species that food neophobia can be subdivided by type of fear: of the object, which can be further split into reactivity to the food’s appearance or presentation, and of consuming or incorporating new food into a diet (“dietary wariness”) [7]. The effect of presentation familiarity on response to novel feed is thus important to accurately interpret food neophobia. For example, animals can be tested in pens that use different methods or positions of food placement than what they were previously familiar with, which could induce object neophobia ahead of food neophobia [49]. Evaluating response to the presentation method (e.g. latency to approach an empty bucket, [3]) separate from the feed, or incorporating the novel presentation into general pen acclimation [47], is important to appropriately isolate neophobia directed at the food itself. Neophobia is known to be reduced when animals are exposed to novel items, like a bucket, repeatedly [13, 15, 50], or if the novel item has familiar components [6], like forage in both hay and TMR, explaining the lower responses in both Pipe and Bucket calves compared to Control.

Calves exhibited transient neophobia, seemingly overcoming both object neophobia and dietary wariness quickly, suggesting they were motivated to consume forage. Most startles, flinches, and retreats occurred within the first few minutes of testing, and all calves began eating within this same time frame. Domestic species are generally less reactive to novelty than their wild correlates [51, 52], possibly explaining the transient response we found. However, this short-lived neophobia is particularly notable in Control calves, who had been previously deprived of forage. While they had a longer latency to start eating TMR compared to calves fed hay, they still began eating within 1 min. This was shorter than in other food neophobia tests in individually housed calves using novel forage (e.g. 60 s vs. 334 s, [3, 47]). In these other neophobia studies, all calves had access to forage in the rearing period [3, 47], possibly contributing to their slower approach compared to our Control group. Control calves also consumed as much TMR as both Bucket and Pipe calves within the first 30 min of having access to this novel food, and persisted in eating novel TMR for most of the test period (Fig 3), despite having continued access to familiar grain. In comparison, Bucket and Pipe calves stopped eating TMR earlier. Motivation for forage, as seemingly demonstrated here, is apparent in calves from a young age. Abnormal behaviors, which are indicative of welfare concerns [53], are reduced by hay provision [19, 32, 33]. Calves will also consume 15–20% of their diet in the form of hay when given the choice [19, 26, 31]. Older age classes of cattle will push weighted gates to access forage [54], even when a grain-based diet is freely available, similar to how Control calves persisted in eating TMR even while engaging in startle behaviors. Taken together, access to forage appears to be important for cattle, and calves that have been deprived of opportunities to consume this seem motivated to access forage as soon as it is provided to them.

Calves with experience consuming hay (Bucket, Pipe) were more efficient at processing novel TMR than inexperienced calves. Calves of all treatments consumed the same amount of TMR, but Bucket calves did this in half the time it took Control calves. This improved processing ability is seen in other settings as forage-exposure time increases, with calves spending half as much time eating as they age despite having access to the same amount of feed (e.g. 13 vs. 6% of observations eating in wk 2 vs. 13, [55]; 30 vs. 14% in month 1 vs. 4, [56]). Consuming forage involves more oral and digestive processing than grain due to the higher fiber content [25–27], so calves fed hay were likely more experienced in these behaviors and better able to transition to consuming a comparable fibrous feed (TMR). Calves fed hay are also reported to consume larger amounts of grain in the preweaning period and TMR in the week following weaning [19, 26, 34], which could further suggest they have more efficient food processing skills than calves reared with only grain. Feeding behaviors and preferences developed in early life can persist beyond the weaning period [57, 58] which may also explain the fast adaptation to TMR by calves fed hay. Pipe calves only tended to consume TMR faster than Control calves. This could reflect an additional layer of adjustment for the Pipe calves as they may have developed different processing behaviors than Bucket calves during the milk-fed period. The pipe feeder encouraged calves to extract hay from a hole at head-height with their tongue and teeth; switching to eating it head-down from an open bucket may have required some adjustment and learning. Overall, exposure to fibrous feeds early in life may facilitate timely development of feeding skills which can assist in the rapid switch to processing novel TMR.

## Conclusion

Early access to hay improves adaptability to novel feed. Dairy calves that had previously consumed hay showed less neophobia to a novel forage than forage-naïve calves, included shorter latencies to begin eating and fewer startles, flinches, and retreats. This reaction was affected by familiarity with forage presentation (i.e. in a bucket or a PVC pipe feeder). Calves that were experienced with hay were also more efficient at processing TMR than naïve calves. Opportunities to process forage appear important to calves: naïve calves exhibited transient neophobia and consumed as much TMR in the test period as forage-experienced calves, suggesting they are motivated to access this feed source.

## Supporting information

S1 Video Example response to novel TMR from a Bucket calf. (MP4)

S2 Video Example response to novel TMR from a Pipe calf. (MP4)

S3 Video Example response to novel TMR from a Control calf. (MP4)

S1 Table Back-transformed model-predicted means, SE, and 95% CI for all outcomes scored, along with model outputs. (PDF)

S1 Fig Photo of starter grain, long chop of mountaingrass hay, and TMR fed to calves. (PDF)
