# Supplementary material for: Response to novel feed in dairy calves is affected by prior hay provision and presentation method
Source: PLoS One. 2023 May 3;18(5):e0284889. doi: 10.1371/journal.pone.0284889 (PMC10155978; doi:10.1371/journal.pone.0284889)
Supplement: S1 Table — (PDF) [file pone.0284889.s004.pdf]

S1 Table. Back-transformed model-predicted means, SE, and 95% CI for all outcomes scored, along with model outputs

Back-transformed model predicted means (obtained using type="response" in emmeans in R) and CI:

| | **Control** | | **Pipe** | | **Bucket** | |
|---|---|---|---|---|---|---|
| **Behavior** | **Emmeans (SE)** | **95% CI** | **Emmeans (SE)** | **95% CI** | **Emmeans (SE)** | **95% CI** |
| Latency to eat TMR (s) | 60.2 (15.7) | [39.0,131.4] | 38.4 (10.6) | [24.4,90.3] | 4.6 (1.3) | [2.9,10.8] |
| Proportion of time spent eating TMR | 0.57 (0.06) | [0.45, 0.69] | 0.38 (0.06) | [0.26,0.50] | 0.26 (0.06) | [0.15,0.36] |
| TMR consumed (g) | 44.7 (9.9) | [30.7,82.3] | 30.9 (6.8) | [21.2,56.8] | 56.0 (13.1) | [37.7,108.5] |

Model results:

Pairwise comparisons and p-values are only presented if the overall effect is significant

| | | **Anova (Type II SS)** | | |
|---|---|---|---|---|
| **Behavior** | **Model type** | **DF** | **chisq** | **p-value** |
| Latency to eat TMR | Generalized linear model | 2,22 | 35.525 | <0.001 |
| Control v. Bucket | | - | - | 0.008 |
| Control v. Pipe | | - | - | 0.511 |
| Bucket v. Pipe | | - | - | 0.012 |
| Proportion of time spent eating TMR | Betaregression | 2,21 | 12.952 | 0.002 |
| Control v. Bucket | | - | - | <0.001 |
| Control v. Pipe | | - | - | 0.070 |
| Bucket v. Pipe | | - | - | 0.300 |
| TMR consumed | Generalized linear model | 2,23 | 3.450 | 0.178 |

Chi-square data + model results:

| | **Control** | **Pipe** | **Bucket** | | | |
|---|---|---|---|---|---|---|
| **Behavior** | **# of calves** | **# of calves** | **# of calves** | **DF** | **X-squared** | **p-value** |
| Startles, flinches, retreats | 9 (100%) | 6 (75%) | 2 (25%) | 2 | 11.213 | 0.004 |
| Lying down | 2 (25%) | 6 (75%) | 4 (50%) | 2 | 4.000 | 0.135 |
