# Supplementary material for: Response to novel feed in dairy calves is affected by prior hay provision and presentation method
Source: PLoS One. 2023 May 3;18(5):e0284889. doi: 10.1371/journal.pone.0284889 (PMC10155978; doi:10.1371/journal.pone.0284889)
Supplement: S1 Fig — (PDF) [file pone.0284889.s005.pdf]

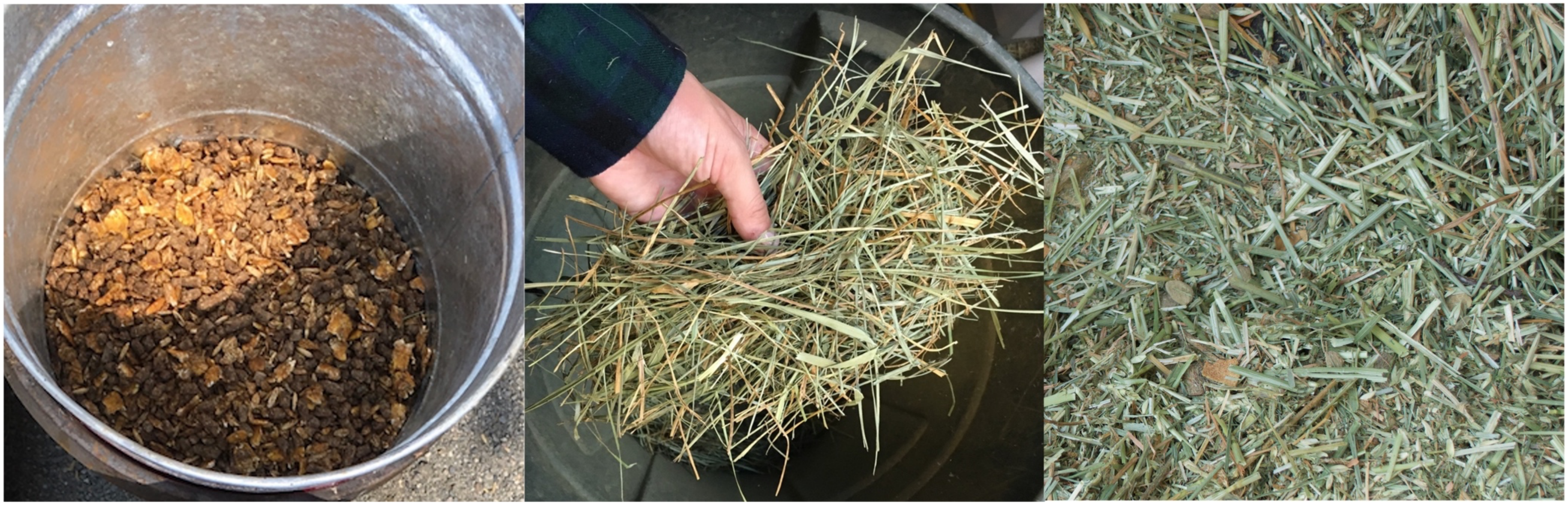

S1 Figure. Starter grain (Starter Calf Feed 901033, Associated Feed and Supply Co.; left), long chop (~19 cm) of mountaingrass hay (center), and total mixed ration (TMR; alfalfa, almond hulls, cottonseed, corn, barley, beet pulp) fed to calves. Dairy calves either received grain, or grain and hay, from birth through weaning. TMR was provided to all calves at the start of weaning (d 50).
